# Trends in regional outpatient antibiotic prescription data and interventions in the Dutch-German EURSAFETY HEALTH-NET-project

**DOI:** 10.1186/1753-6561-5-S6-P144

**Published:** 2011-06-29

**Authors:** A Jurke, M Flume, R Köck, AW Friedrich

**Affiliations:** 1 Infectiology and Hygiene, NRW Institute of Health and Work, Münster, Germany; 2 Prescription Management, Association of Statutory Health Insurance Physicians Westphalia-Lippe, Dortmund, Germany; 3 Institute of Hygiene, University Hospital, Münster, Germany; 4 Department of Medical Microbiology, University Medical Centre , Groningen, Netherlands

## Introduction / objectives

Increasing prescription of broad-spectrum antibiotics is regarded to facilitate selection of multiresistant germs. Surveillance of outpatient antibiotic use might contribute to prevent the spread of Methicillin-resistant *S. aureus* (MRSA) within the EURSAFETY HEALTH-NET. Training sessions on MRSA and antibiotic awareness were offered to general practitioners. A possibility for reimbursement of MRSA eradication therapy in outpatients has been created.

## Methods

Regional data for outpatient prescription of antibiotics (based on Defined Daily Doses (DDD)) were collected by the Association of Statutory Health Insurance Physicians Westphalia-Lippe (KVWL) and analyzed for the years 2002 to 2009. In order to compare the prescription of different antibiotics like fluoroquinolones or mupirocin in the EUREGIO to the whole KVWL region, we used the Cochrane Armitage Trend Test.

## Results

Altogether, a total of 12.2 DDD / day per 1,000 inhabitants (DID) were prescribed in 2002, followed by 12.9 DID, 12.9 DID, 14.4 DID, 13.8 DID, 14.4 DID, 14.7 DID and 14.8 DID from 2003 to 2009, respectively. From 2002 to 2009 the percentage of prescriptions of all antibiotics and of fluorochinolones decreased significantly in EUREGIO relating to the whole KVWL region. In contrast, the number of mupirocin prescriptions increased significantly more in EUREGIO than in KVWL region.

## Conclusion

As desirable, EUREGIO tends to prudent antibiotic use. The increasing number of mupirocin prescription among outpatients reflects the growing demand and, facilitated by new refunding possibilities, the increasing implementation of MRSA eradication therapy in outpatient care.

## Disclosure of interest

None declared.

